# Case Report: Extrarenal *TFE3* fusion-related renal cell carcinoma

**DOI:** 10.3389/fonc.2025.1592042

**Published:** 2025-05-29

**Authors:** Zhouliang Yang, Ting Li, Kejun Lv, Xiaowei Zhang

**Affiliations:** ^1^ Department of Pathology, The First People’s Hospital of Yongkang, Yongkang, Zhejiang, China; ^2^ Department of Imaging Medicine, The First People’s Hospital of Yongkang, Yongkang, Zhejiang, China; ^3^ Department of Pathology, Affiliated Dongyang Hospital of Wenzhou Medical University, Dongyang, Zhejiang, China

**Keywords:** renal cell carcinoma, *TFE3* fusion, extrarenal, pathology, surgery

## Abstract

**Introduction:**

Transcription factor binding to IGHM enhancer 3 (*TFE3*) fusion-related renal cell carcinoma (*TFE3*-RCC) is a rare subtype of RCC. Its pathogenesis is primarily associated with chromosomal translocations resulting in *TFE3* fusions. *TFE3*-RCC is most commonly observed in adolescents and young adults, with a higher incidence in women than in men. Typically, *TFE3*-RCC initially presents as painless hematuria, an abdominal mass, or with systemic symptoms. In recent years, with advancements in molecular diagnostic techniques, the diagnosis rate of *TFE3*-RCC has increased. However, extrarenal occurrences of *TFE3*-RCC remain rare. *PRCC* can fuse with *TFE3* causing *PRCC*-*TFE3* fusion-related RCC, a unique subtype of *TFE3*-RCC.

**Case presentation:**

We report a case of *PRCC*-*TFE3* RCC in a 29-year-old woman who was hospitalized owing to a mass in her upper abdomen. To our knowledge, this is the second reported instance of an extrarenal occurrence. Imaging revealed a large mass in the left retroperitoneum, and postoperative pathology revealed that the tumor cells were either epithelioid- or spindle-shaped, with large nuclei, prominent nucleoli, and abundant chromatin. The cells were densely arranged in nests or sheets, with abundant eosinophilic or amphophilic cytoplasm. Immunohistochemical analysis revealed diffuse and strong nuclear positivity for *TFE3* but negativity for carbonic anhydrase IX (CAIX). Fluorescence *in situ* hybridization did not detect a *TFE3* break, but RNA sequencing confirmed the presence of a *PRCC-TFE3* fusion.

**Conclusion:**

The diagnosis of *TFE3*-RCC requires a comprehensive evaluation of histological features, immunohistochemical markers, and molecular testing. *PRCC*-*TFE3* RCC is highly aggressive with a high recurrence rate and poor prognosis in adults. Surgical resection is the primary treatment for localized lesions. However, close follow-up is necessary owing to a high risk of recurrence and metastasis. Targeted therapies and immunotherapies are potential treatment options for patients with advanced or metastatic disease.

## 1 Introduction

Renal cell carcinoma (RCC) is one of the most common malignant tumors of the urinary system, and its incidence has recently increased worldwide (1). In recent years, with rapid advancements in molecular biology techniques, our understanding of the pathogenesis, molecular characteristics, and clinical presentation of RCC has expanded (2). Transcription factor binding to IGHM enhancer 3 (*TFE3*)-RCC is a unique subtype of RCC that has garnered increasing attention owing to its distinct molecular features and clinical manifestations (3). *TFE3* fusion-related RCC (*TFE3*-RCC) is most commonly observed in adolescents and young adults, with a higher prevalence in women than in men. Clinically, patients often present with painless hematuria, abdominal masses, or systemic symptoms (such as weight loss and fever) as initial manifestations. Some patients may experience organ dysfunction attributed to tumor metastasis. Although *TFE3*-RCC is relatively rare, its unique molecular features and clinical manifestations play a significant role in the diagnosis and treatment of RCC. Herein, we report on the clinical and pathological characteristics of a patient with *PRCC-TFE3* fusion-related RCC treated at our hospital, aiming to provide a reference for its management in clinical setting.

## 2 Case description

A 29-year-old woman was admitted to the First People’s Hospital of Yongkang on September 2, 2024, with a chief complaint of upper abdominal bloating and discomfort for more than 3 months. The patient began experiencing these symptoms without any obvious triggers, such as overeating. She did not experience nausea, vomiting, poor appetite, or fatigue. Her symptoms did not improve significantly, and she noticed a palpable mass in the upper left abdomen measuring approximately 15 × 10 cm, with mild tenderness on palpation. The mass exhibited relatively clear borders, moderate mobility, and mild tenderness. The rest of the abdomen demonstrated no significant or rebound tenderness; bowel sounds were normal. No edema was observed in the lower extremities.

The patient’s medical and family history revealed no abnormalities, such as past medical conditions, relevant familial cancers or genetic conditions. She underwent preoperative laboratory testing, including routine blood tests; urinalysis; and renal function, liver function, and adrenal function analyses. All preoperative laboratory test results were within normal limits.

### 2.1 Imaging examinations

#### 2.1.1 Abdominal computed tomography

**Figure 1:**
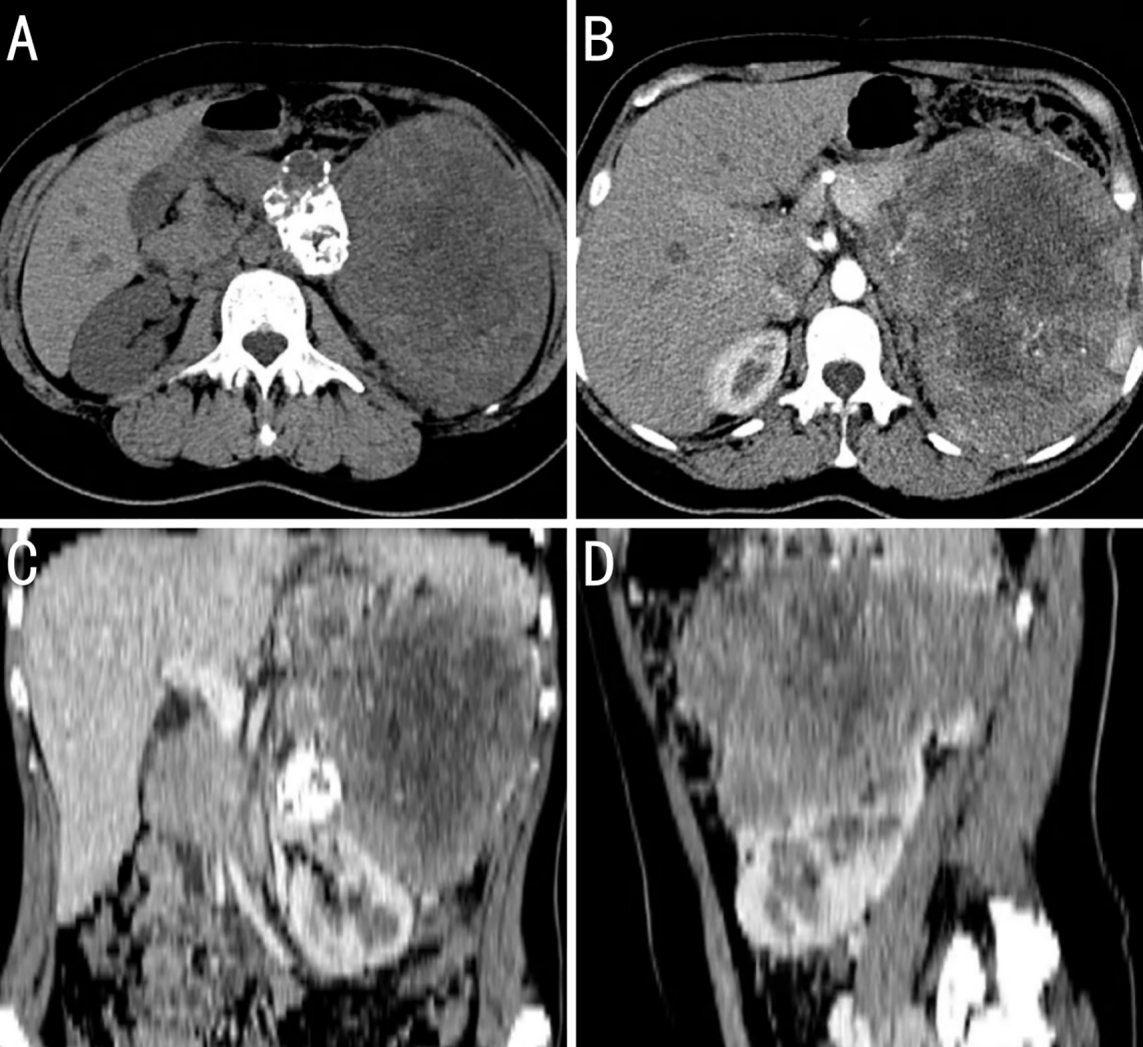
Computed tomography (CT) image findings of the case. **(A)** The tumor has a slightly higher density at the periphery and a lower density in the center, with possible patchy calcifications. **(B)** During the arterial phase of the contrast-enhanced scan, the feeding artery of the tumor is traced back to the abdominal aorta, not the renal artery, which suggests that the tumor is of extrarenal origin; there is extensive necrosis within the tumor that shows no enhancement, while the solid areas show moderate enhancement; and the body and tail of the pancreas are obscured by the tumor invasion. **(C)** Coronal reconstruction images from the CT scan show that the outline of the kidney is intact. **(D)** Sagittal reconstruction images from the CT scan show that the tumor encases the splenic vessels.

The patient underwent abdominal computed tomography. A 126 × 153 mm mixed-density mass with local calcification was observed in the left retroperitoneum. An enhanced scan revealed marked heterogeneous enhancement, and the lesion exhibited unclear boundaries with the caudal part of the peritoneum. The left adrenal gland was poorly visualized. Curved venous shadows were observed around the lesion, with local narrowing of the splenic vein. The splenic artery was well visualized, with no local stenosis or dilation. The left kidney was significantly compressed and displaced downward. The liver revealed no enlargement, with uniform density and smooth margins. The gallbladder was not enlarged, with smooth walls and no thickening. The spleen was enlarged, with smooth margins and no abnormal density foci. The left kidney demonstrated punctate high-density shadows. The right adrenal gland revealed no abnormalities in its shape, size, or density. A small amount of fluid was observed in the pelvis, and no enlargement of the retroperitoneal lymph nodes was observed. Based on the abdominal computed tomography, the patient was diagnosed with a large mass in the left retroperitoneum that possibly originated from the tail of the pancreas as a solid pseudopapillary neoplasm or a germ cell tumor (**Figures 1A–D**).

#### 2.1.2 Abdominal magnetic resonance imaging

**Figure 2:**
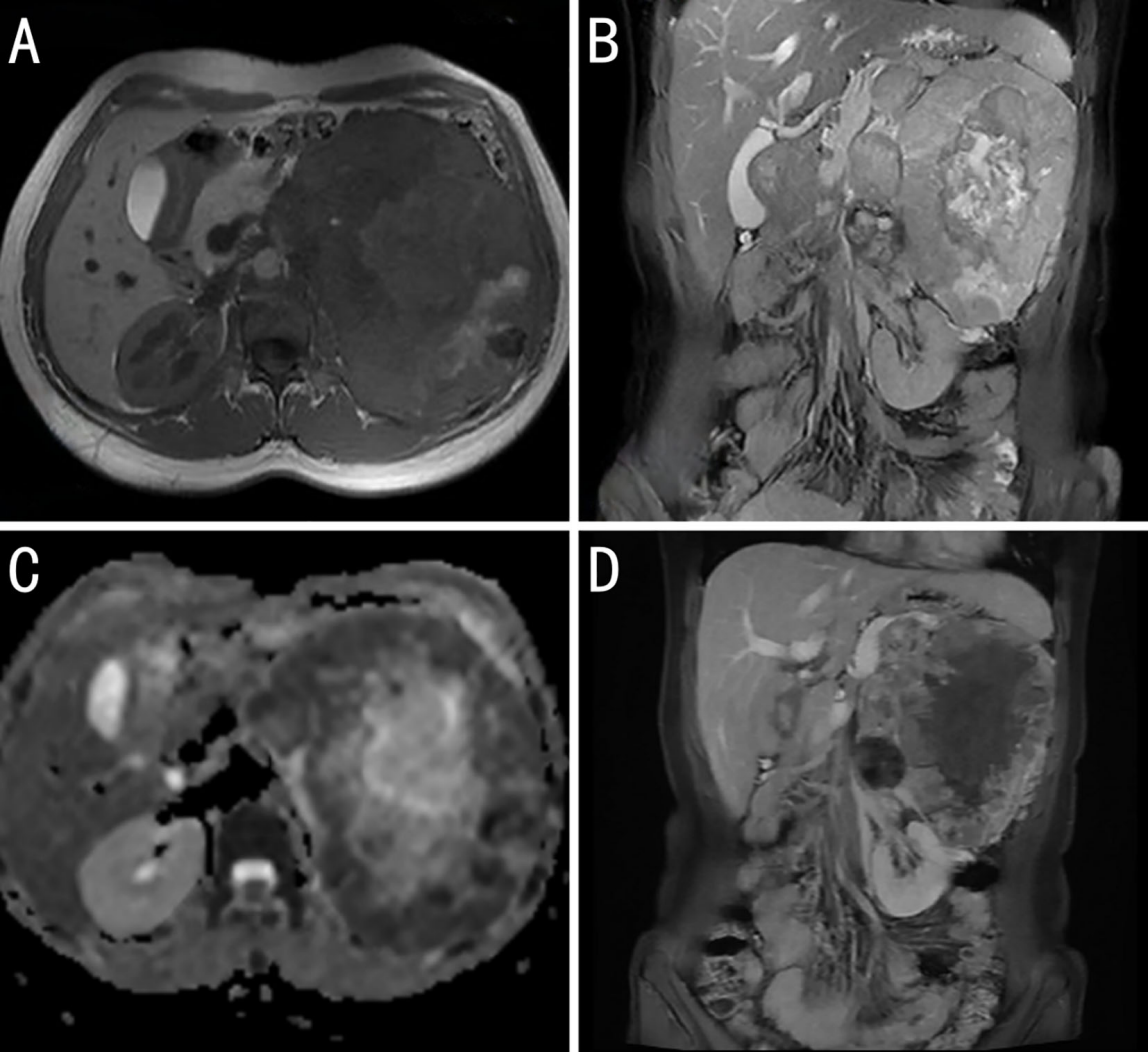
Magnetic resonance imaging (MRI) image findings of the case. **(A)** T1 weighted imaging: The body and tail of the pancreas are obscured by tumor invasion. **(B)** T2 fat-suppressed coronal images show mostly slightly high signal intensity (with high signal intensity in the center, indicating necrosis) and a low signal ring at the periphery. **(C)** Apparent diffusion coefficient (ADC): The peripheral solid portions show low signal intensity. The high signal intensity on diffusion weighted imaging and low signal intensity on ADC suggest restricted diffusion within the tumor, which is related to the dense arrangement of tumor cells. **(D)** Contrast-enhanced coronal images show tumor invasion of the body and tail of the pancreas, narrowing of the splenic vein, and varices in the gastric fundus, suggesting pancreatic splanchnic portal hypertension.

The patient underwent abdominal magnetic resonance imaging. A mass measuring approximately 125 × 153 mm, with mixed high and low signals on T1-weighted imaging (T1WI) and heterogeneous high signals on T2-weighted imaging (T2WI), was visible in the left retroperitoneum. Local diffusion restriction was observed, and the enhanced scan revealed marked heterogeneous enhancement. The lesion had unclear boundaries with the pancreatic tail, and multiple dilated veins were observed around the mass. The left adrenal gland was poorly visualized, and the left kidney was significantly compressed and displaced downward. The liver had a normal shape and size, with no obvious abnormal signal foci on T1WI or enhanced scans. The intrahepatic bile ducts and gallbladder revealed no significant dilatation. The spleen was enlarged. The pancreas had normal morphology and signal intensity. The right adrenal gland showed no abnormalities in its shape, size, or density. The retroperitoneal lymph nodes were not enlarged. Small, round T2WI hyperintense foci with clear margins were observed in both kidneys, the largest being approximately 6 mm; no enhancement was observed. Based on the magnetic resonance imaging findings, the patient was diagnosed with a large mass in the left retroperitoneum that was possibly a solid pseudopapillary neoplasm of the pancreas, originating from the pancreatic tail, or a germ cell tumor (**Figures 2A–D**).

### 2.2 Surgical procedure

**Figure 3:**
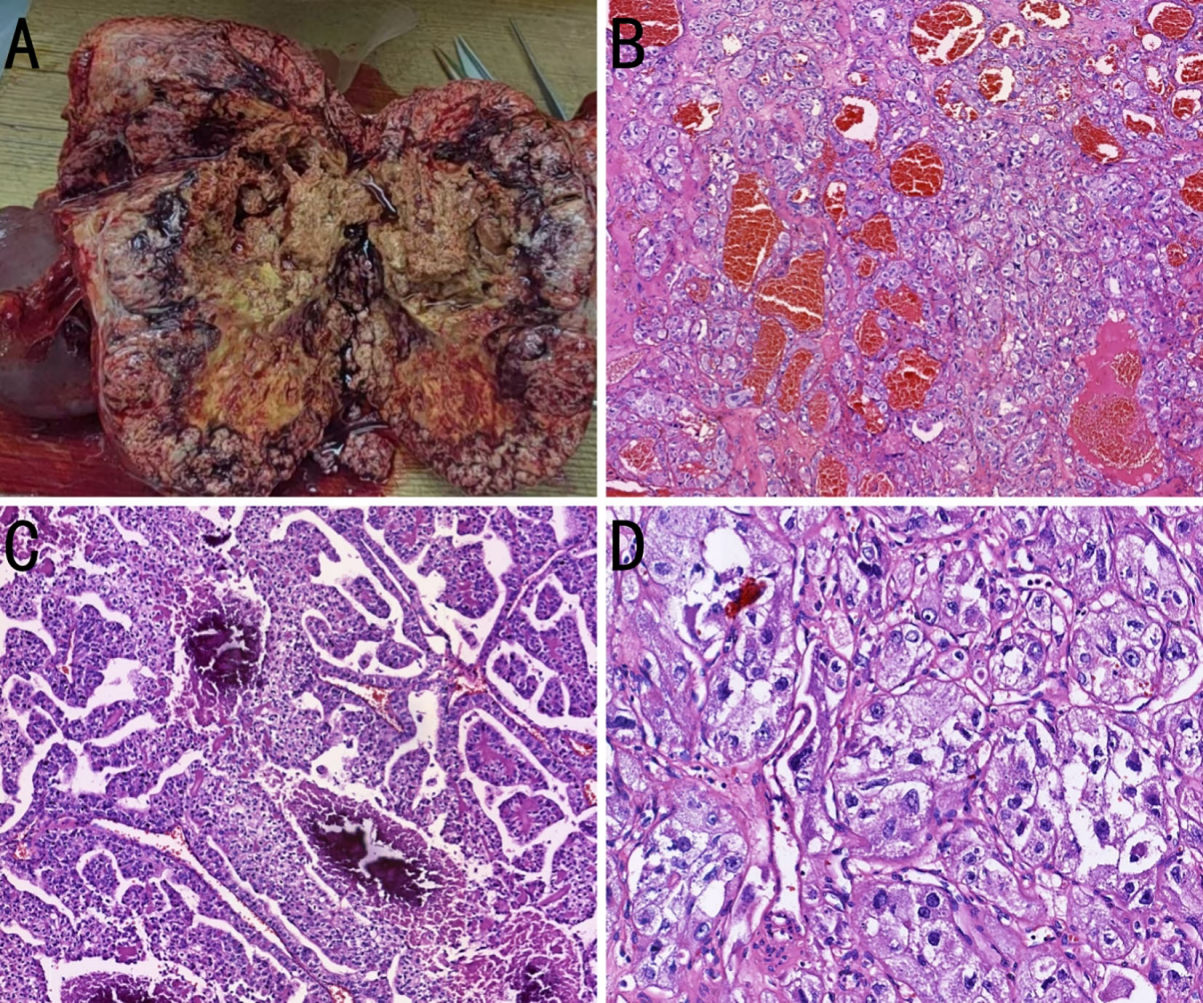
Pathological images of the case. **(A)** A large gross tumor is observed, with incision specimen surface displaying a grayish-red and grayish-yellow multicolored appearance. The tissue texture is soft and accompanied by necrosis. **(B)** The tumor cells are arranged in tubular, papillary, and nested patterns, with abundant blood sinusoids (hematoxylin and eosin [H&E], ×50). **(C)** Necrosis and calcification are observed within the tumor tissue (H&E, ×100). **(D)** The tumor cells exhibit atypia, with cytoplasm that is either clear or eosinophilic, and prominent nucleoli (H&E, ×200).

During the preoperative multidisciplinary discussion, the experts unanimously agreed that, based on the preoperative imaging results showing no distant metastasis, surgical resection of the retroperitoneal tumor is the preferred treatment option for this case. On September 12, 2024, the patient underwent resection of the retroperitoneal tumor, total splenectomy, distal pancreatectomy, and left adrenalectomy. The resected specimen was a large, encapsulated mass measuring 17 × 15 × 10 cm. On incision specimen, it exhibited a heterogeneous gray-red and gray-yellow appearance, soft texture with necrosis, and nodular protrusions. The protruding areas exhibited calcification and cystic changes. The adherent adrenal gland measured 4 × 2.5 × 1 cm. The splenic tissue adjacent to the mass measured 12 × 9 × 2.5 cm, and a small amount of pancreatic tissue was also resected, measuring 3 × 2.5 × 1 cm. Postoperative pathology of the mass was performed (**Figures 3A– D**).

### 2.3 Postoperative pathology

**Figure 4:**
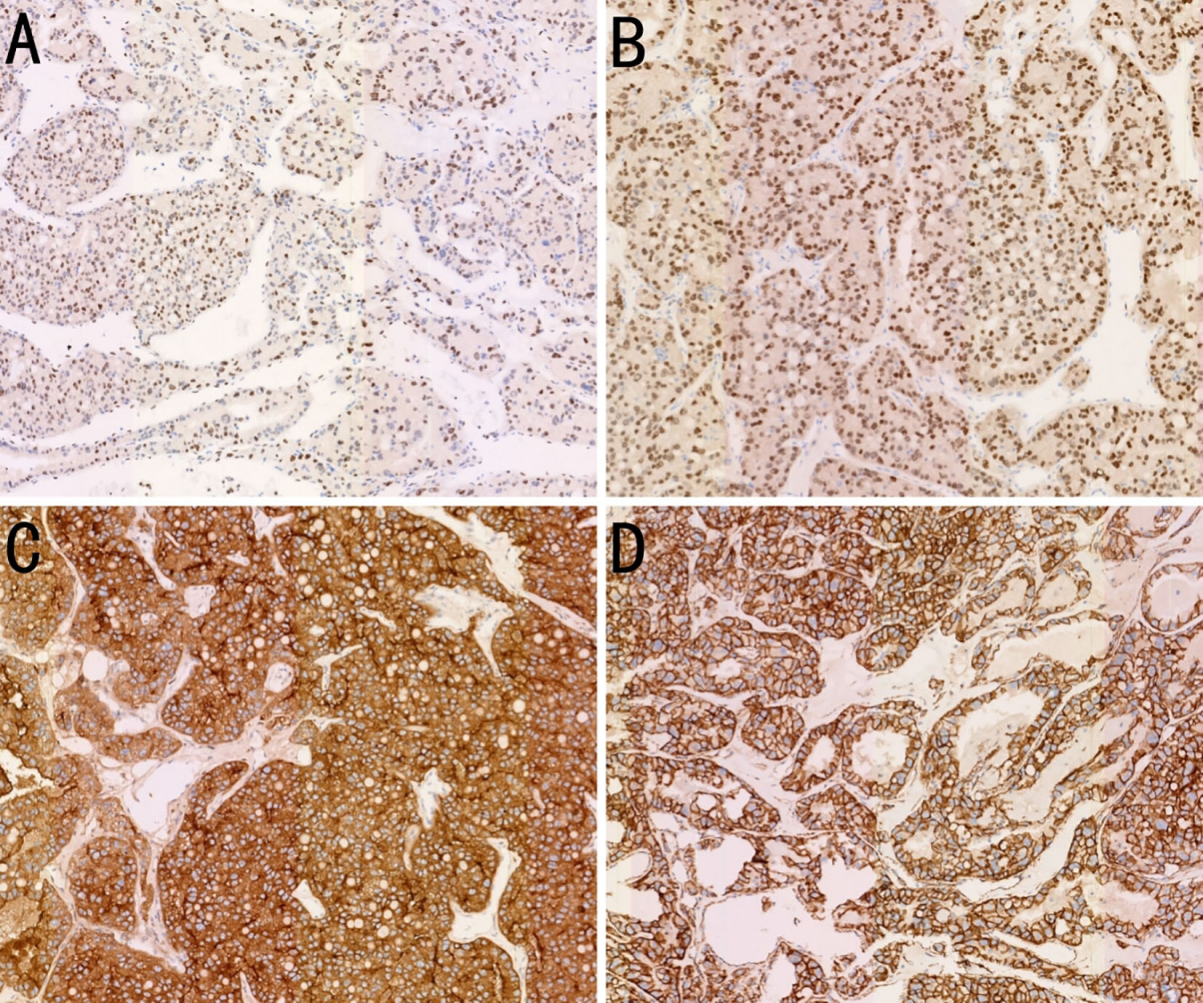
Immunohistochemistry (IHC) findings of the case. **(A)** Expression of transcription factor binding to IGHM enhancer 3 (*TFE3*) in the tumor tissue (IHC, ×100). **(B)** Expression of PAX8 in the tumor tissue (IHC, ×100). **(C)** Expression of CD10 in the tumor tissue (IHC, ×100). **(D)** Expression of β-catenin on tumor tissue membranes (IHC, ×100).

Microscopically, the tumor cells were uniform in size, with abundant cytoplasm, some of which appeared clear. The nucleoli were prominent, and a few mitotic figures were observed. The cells were arranged in tubular, papillary, or nested patterns. The tumor had abundant blood sinusoids. Calcification and ossification were observed in some areas. The surrounding capsule was thick and intact, with fibrovascular proliferation. Fibrous adhesion to the adrenal gland, kidney, and pancreas were present, but no invasion was observed. Immunohistochemical staining was negative for CK7, PAX8, CGA, SYN, S100, ALK, CD56, MelanA, carbonic anhydrase IX (CAIX), HMB45, Arg1, SALL4, PR, inhibin, and SF-1. The tumor was partially positive for CD117, CD10, and vimentin, and positive for *TFE3*, membrane β-catenin, and cyclin D1 (**Figures 4A–D**). Fluorescence *in situ* hybridization revealed no evidence of *TFE3* breakage or rearrangement. RNA sequencing revealed a *PRCC-TFE3* fusion, suggestive of RCC with *TFE3* translocation. The patient was diagnosed with *PRCC-TFE3* fusion-related RCC based on the pathological findings.

### 2.4 Postoperative follow-up

The patient experienced no recurrence or distant metastasis during 6 months of postoperative follow-up.

## 3 Discussion

*TFE3*-RCC is a subtype of Xp11.2 translocation/*TFE3* fusion RCC that belongs to the MiT family of translocation RCCs (4). *TFE3*-RCCs are relatively rare; however, in recent years, with advancements in molecular diagnostic techniques, their diagnostic rate has considerably increased. In terms of tissue origin, *TFE3*-RCC typically arises from renal tubular epithelial cells. In our case, the patient with *PRCC*-*TFE3* fusion-related RCC exhibited no evidence of renal parenchymal involvement based on imaging and gross surgical observations. However, the tumor was located in the extrarenal retroperitoneal region. To our knowledge, this is the second reported case of *PRCC-TFE3* fusion-related RCC occurring outside the kidney (5). Several potential tissue origins of extrarenal renal tumors have been reported (6–8). First, tumors occurring in the retroperitoneal area around the kidneys are generally presumed to originate from the ectopic remnants of the metanephric blastema, which fail to differentiate and mature properly, leading to neoplastic proliferation. Second, tumors occurring in the ovaries, uterus, vagina, testes, and inguinal regions presumably arise from remnants of the mesonephric duct, with evidence of mesonephric duct remnants found in the tumor tissue. Third, tumors in the mediastinum and chest wall may originate from remnants of the pronephros. Fourth, tumors occurring in locations distant from the urogenital system that contain tissue components with multilineage differentiation potential are likely derived from embryonic stem cells. In our case, the *PRCC-TFE3* fusion-related RCC occurred in the retroperitoneal region. It is speculated that it may originate from the ectopic remnants of the metanephric blastema.

*TFE3*-RCC has certain characteristic clinical manifestations; however, these overlap with other subtypes of RCC (9). *TFE3*-RCC is most prevalent in children and adolescents and has a relatively high incidence in women. The most common clinical symptom is a painless renal mass, whereas the classic triad of renal cancer (hematuria, flank pain, and an abdominal mass) is relatively rare. Additionally, some patients may present with extrarenal symptoms, such as weight loss, fever, and fatigue, due to tumor metastasis. Imaging examinations often depict the tumor as a low-density or multicystic lesion, with calcification visible in some cases. Owing to the lack of specific symptoms, many patients may not show noticeable symptoms in the early stages of the disease, and tumors are often incidentally discovered during imaging examinations.

The pathological diagnosis of *TFE3*-RCC relies on the integrated application of histological features, immunohistochemical markers, and molecular testing. Microscopically, the tumor cells often exhibit papillary, glandular, or solid nested arrangements. The cytoplasm ranges from eosinophilic to clear, with prominent nucleoli in some areas. Psammoma bodies may be observed in certain cases; moreover, diffuse, strong nuclear positivity for *TFE3* is an important diagnostic marker. Fluorescence *in situ* hybridization and polymerase chain reaction analysis are reliable methods for confirming *TFE3* rearrangements (10, 11). For the differential diagnosis, *TFE3*-RCC should be distinguished from other RCC subtypes with similar morphological features, such as clear cell and papillary RCC. Clear cell RCC typically shows clear cytoplasm and is positive for CAIX in immunohistochemistry, whereas *TFE3*-RCC is negative for CAIX. Additionally, transcription factor EB (TFEB)-rearranged RCC may exhibit similar morphological features but is positive for TFEB and cathepsin K in immunohistochemistry. Therefore, combining immunohistochemistry and molecular test results is crucial for the accurate diagnosis of *TFE3* fusion-related RCC (12).

The prognosis of *TFE3*-RCC is relatively poor, especially in adult patients, who tend to have a worse prognosis than those with clear cell RCC. Distant metastasis, older age, and presence of inferior vena cava tumor thrombus are considered adverse prognostic factors in *TFE3*-RCC (13). The treatment for *TFE3*-RCC is similar to that for other RCC subtypes and primarily includes surgical resection, targeted therapies, and immunotherapy. Surgical resection is the primary treatment for localized lesions. However, owing to the high aggressiveness and recurrence rates of this subtype, close follow-up is essential after surgery (14). Targeted immunotherapy is an important treatment option for patients with advanced or metastatic *TFE3*-RCC. Recently, immune checkpoint inhibitors have demonstrated efficacy in the treatment of RCC. However, their application in *TFE3*-RCC warrants further clinical research.

In conclusion, the diagnosis of *PRCC*-*TFE3* RCC relies on a comprehensive evaluation combining histological features, immunohistochemical markers, and molecular testing. This rare subtype of RCC is highly aggressive, with a high recurrence rate and poor prognosis in adults. Although surgical resection remains the primary treatment for localized lesions, close follow-up is essential due to the risk of recurrence and metastasis. For patients with advanced or metastatic disease, targeted therapies and immunotherapies may offer potential treatment options.

## Data Availability

The original contributions presented in the study are included in the article/**Supplementary Material**. Further inquiries can be directed to the corresponding authors.
